# Inhibition of RNA-m^6^A Methylation in Breast Cancer Cells Carrying *TP53* R273H Mutant Reactivates Natural Killer Cells for Anticancer Immunity

**DOI:** 10.3390/cimb48101021

**Published:** 2026-10-02

**Authors:** Mohammad N. Amin, Md Saqline Mostaq, Seetharama Jois, Lin Kang, Yong-Yu Liu

**Affiliations:** 1 School of Basic Pharmaceutical and Toxicological Sciences, University of Louisiana at Monroe, Monroe, LA 71201, USA; aminmn@warhawks.ulm.edu (M.N.A.); saqlinemostaq@gmail.com (M.S.M.); 2 Department of Pathobiological Sciences, School of Veterinary Medicine, Louisiana State University, Baton Rouge, LA 70803, USA; sjois@lsu.edu; 3 Department of Biomedical Affairs and Research, Edward Via College of Osteopathic Medicine, Monroe, LA 71203, USA; lkang@ulm.vcom.edu; 4 Center for One Health Research, VA-MD Regional College of Veterinary Medicine, Blacksburg, VA 24061, USA

**Keywords:** p53 tumor suppressor, natural killer cell, missense mutation, *N*^6^-methyladenosine, breast cancer, NKG2D ligands, ULBP1

## Abstract

Oncogenic p53 mutant proteins not only promote cancer cell proliferation but also evade immune destruction for tumor progression. Besides hijacking immune checkpoints, p53 mutants in cancer cells may deactivate anticancer immune cells for evasion. Herein, we report that p53 R273H mutation in human breast cancer cells deactivated natural killer (NK) cells in immune evasion; conversely inhibition of RNA methylation with neplanocin A (NPC) reactivated NK cells and reinforced cytotoxicity against cancer. Human MDA-MB-468 breast cancer cells, which carry homozygous p53 R273H, significantly deactivated NK-92 MI cells, while either MCF-7 cancer cells or MCF-10A noncancerous cells, both are wild-type p53, activated NK cells to produce interferon-γ (IFN-γ) production in co-cultures. Consistently, NK cytotoxicity to tumor organoids induced by MCF-7 cells was approximately 4-fold higher than MDA-MB-468 cells. Interestingly, inhibition of RNA m^6^A methylation of MDA-MB-468 cells with NPC markedly reactivated proliferation of NK-92 MI cells, increased IFN-γ levels and destructed tumor organoids. Further mechanistic study showed that inhibition of RNA m^6^A-modification enhanced p53-associated NKG2D ligands, including ULBP1 in cancer cells, to activate NK cells through NKG2D receptors in antitumor immunity. Altogether, our study suggests that targeting m^6^A methylation in p53 mutant-cancer cells can reactivate NK cell-based antitumor immunity.

## 1. Introduction

Tumor suppressor *TP53* generates protein p53 that stabilizes human genome with respect to propensity for tumorigenesis through modulating transactivation of p53-responsive genes in response to cellular stress [1,2]. Mutation of *TP53* is detected in approximately half of human cancer cases, more frequently (~70%) manifested as full-length missense mutant proteins [3,4]. The most recurrent of these missense mutations (~30%) of p53 (mtp53) are several ‘hotspot mutations’ within the DNA-binding domain, including the most common at codons R175, R248, R273, which have been identified in almost all cancer cases [4,5,6] (http://p53.free.fr/Database/p53_cancer/all_cancer.html). In breast cancer, hotspots of p53 mutation, including R175, G245, R248 and R273 mutants are prevalent and prominently associated with tumor resistance to treatments and worse cancer prognosis [7,8].

Missense mutations of *TP53* (mtp53) often obtain oncogenic gain-of-function (GOF) with mutant proteins in addition to loss the function of wild-type form in tumor suppression. Besides the more common recognized GOF in promoting cell proliferative or anti-apoptosis, recent studies point that inactivation of p53 restrains immune destruction against tumors, and further reactivated cancer cells in the tumor microenvironment (TEM) can remodel the immune responsiveness, offsetting immune evasion and boosting antitumor immunity [9]. Long with promoting tumor growth, GOF of mtp53 by varies types of cancer cells advance TEM, including immune evasion [10,11,12]. mtp53 tumors often display more immune evasive than wtp53 tumors. Cancer cells with p53 mutants cultivate immunoediting ability and evade the immune surveillance and destruction, which mainly rely on T cells and NK cells [6,11,13]. NK cells recognize cancerous cells by a variety of receptors, so as launch potent assaults [14]. NK cell receptors are specific for the ligands that are produced by cancerous cells and entice NK cells to distinguish those cells. The increased expression of activating ligands or the decreased expression of inhibitory ones in cancer cells can activate NK cells for anticancer immunity [15,16]. Activated NK cells secrete various cytokines, including interferon-gamma (IFN-γ) and tumor necrosis factor (TNF), mounting direct cytotoxic attacks, thus destructing tumors [17]. Among these, the NK group member 2D (NKG2D) [18] and DNAX accessory molecule-1 (DNAM-1) [19] are the most important two receptors on NK cells. NKG2D receptor is a lectin-like type-2 transmembrane receptor and is mainly located in NK cells, although is also expressed with very low levels in subtypes of cytotoxic T cells [20]. The family of NKG2D ligands mainly include MHC class I-chain-related proteins A (MICA) and B (MICB), retinoic acid early transcript 1 (Rae1), histocompatibility antigen 60 (H60), and mouse UL16-binding protein-like transcript 1 (Mult1) [18,21]. NKG2D receptors of human NK cells can bind to these MHC I-related cell surface ligands, for example the MICA/ULBP subfamilies of proteins [18]. These NKG2D ligands are often dysregulated in cancer cells, consequently suppressing expression of these stimulatory ligands is one signifies by which aggressive cells escape immune surveillance [22]. Recent studies reported that missense p53 mutants, either p53 R175H in human NSCLC cells or p53 G242A in murine breast cancer cells (corresponding to human p53 G245A) modulated the expression of NKGD2 ligands and deactivated NK cells as well as antitumor immunity; on the other hand, reactivating p53 function boosted NK cells against tumors [10,23,24,25].

*N*^6^-methyladenosine (m^6^A) is the most prevalent RNA modification and regulates the stability and translation of modified mRNAs of eukaryotic cells [26,27,28]. In response to cell stress under treatments, m^6^A is dynamically regulated by its writer, methyltransferase components (METTL3, METTL14, and WTAP), erasers (demethylase, FTO, ALKBH5). The m^6^A readers, a group of RNA-binding proteins that recognize and bind to m^6^A-modified transcripts can mediate RNA metabolism and RNA splicing to determine the fate of mRNA in translation [29,30]. Recent studies show that m^6^A modification can modulate GOF of p53 mutants and cancer drug resistance [2,31,32]. In present study, we seek whether m^6^A modification in breast cancer cells carrying p53R273 mutant modulates NK cell-based anticancer immunity.

## 2. Materials and Methods

### 2.1. Cell Lines and Cell Culture

Human breast adenocarcinoma MDA-MB-468 cells (*TP53* R273H^+/+^; ER^−^) [33,34], MCF-7 (wild-type TP53) breast cancer cells and MCF-10A cells (noncancerous mammary epithelial, ER^+^, wild-type p53) were purchased from ATCC (Manassas, VA, USA). MDA-MB-468 and MCF-7 cells were maintained in Dulbecco’s-modified Eagle’s medium (DMEM, Invitrogen) supplemented with 10% fetal bovine serum (FBS) and 100 units/mL penicillin, 100 µg/mL streptomycin, and 584 mg/liter l-glutamine.

Cells of MCF-10A were maintained in DMEM/Ham’s F12 medium containing 10% FBS, epidermal growth factor (20 ng/mL), hydrocortisone (500 ng/mL), cholera toxin (100 ng/mL), insulin (5 μg/mL), penicillin and streptomycin (concentrations as mentioned above). Human natural killer cells, NK-92 MI (wt. TP53, NKG2D^+^) [35] were purchased from ATCC. NK-92 MI cells were cultured in NK culture medium, which is MyeloCult H5100 medium (Catalog#05150 STEMCELL, Vancouver, BC, Canada) containing hydrocortisone (1 μM), penicillin, streptomycin, and *L*-glutamine (as mentioned above). All of these cells were cultured in a humidified incubator with 95% air and 5% CO_2_ at 37 °C, and routinely passaged per 2 or 3 days. (-)-Neplanocin A (NPC, an inhibitor of *S*-adenosylhomocystein hydrolase) (Catalog#10584) [2,36], and doxorubicin hydrochloride (Dox) both were purchased from Cayman Chemical (Ann Arbor, MI, USA).

### 2.2. NK Cell Labelling and Co-Culture

NK-92 MI cells (passages 3–10; 1 × 10^6^ cells) were labelled by incubation with 5 μM CellTrace^TM^ CFSE (Catalog# C34554 Thermo Fisher, Carlsbad, CA, USA) in 1 mL PBS solution at 37 °C for 20 min. The labelled NK-92 MI cells (2.5 × 10^4^ cells) in NK culture medium were placed into 24-well plate alone (service as controls) or with cancer cells (E/T ratios 1:0.5, 1:1). After 4 h, doxorubicin (Dox, 200 nM final concentration) were added and continuously co-cultured for additional 48 h. Images of co-culture were captured using an EVOS FL cell imaging system with color CCD camera (200× magnification). The cells were harvested with trypsin-EDTA with centrifugation for immediate flow cytometry analysis. The co-cultured media were collected and stored at −80 °C for further assays.

### 2.3. Quantitative Analyses of NK-92 MI Cells Using Imaging Flow Cytometry (IFC)

The assessments of labelled NK cells with IFC were performed as described previously with minor modification [10,37]. Briefly, the treated cells were harvested with Trypsin-EDTA and resuspended in 1% BSA PBS solution (1 × 10^5^ cell/mL). Each sample of these (8000 events, triplicate; 40× magnification) were acquired on ImageStreamX Mark II imaging cytometer (EMD Millipore, Burlington, MA, USA) and further analyzed using Image Data Exploration and Analysis Software (IDEAS^®^ 6.2, EMD Millipore). An analysis algorithm was established and validated by microscopy images to gate events based on area, intensity, and aspect ratio (length:width). A log scale was used for the side scatter area (SSC-A) profile for discriminating flow-count of fluorophores from NK cells in samples [38].

### 2.4. ELISA of Interferon-Gamma (IFN-γ)

This assay was performed using human IFN-gamma ELIS kit (RayBiotech, Peachtree Corners, GA, USA, Cat#PO1579), following the kit instruction. Briefly, the collected medium (25 μL + 75 μL dilution buffer/well) were incubated in the Human IFN-γ Microplate and then with Human IFN-γ Conjugate. The optical density of converted tetramethylbenzidine (TMB) were measured at 450 nm using Synergy HT microplate reader (Marshall Scientific, Hampton, NH, USA). IFN-γ levels of samples were quantitated against human IFN-γ standard curves with dilution factor.

### 2.5. Organoid Co-Culture

It was conducted as described previously with minor modification [38,39,40]. Cells of MDA-MB-468 or MCF-7 lines (passages 3–10; 1 × 10^6^ cells) were labelled by incubation with 5 μM CellTrace^TM^ CFSE (Catalog# C34554, Thermo Fisher Scientific, Carlsbad, CA, USA) in 1 mL PBS solution at 37 °C for 20 min. The labelled cancer cells (2000 cells) in 10% FBS DMEM medium were placed on matrigel (Catalog#CB-40234, Thermo Fisher Scientific) in 96-well ultra-low binding and U-shaper bottom microplates (Catalog#12-456-721S24) and cultured 2 days to form organoids. NK-92 MI cells in 10% FBS DMEM medium containing 25 μg/mL propidium iodide (PI, Thermo Fisher Scientific) were added in the experimental organoid wells and co-culture with tumor organoids for additional 72 h. Images of NK-organoids were captured using an EVOS FL cell imaging system with color CCD camera (40× magnification; Thermo Fisher Scientific). The mean fluorescent intensity (MFI) of PI were assessed using ImageJ program (https://imagej.net/ij/). Cytotoxic activity of NK-92 MI cells was calculated using the formula: Specific killing (%) = (Experimental MFI-−Spontaneous MFI)/(Maximum MFI−Spontaneous MFI) × 100.

### 2.6. ELISA of RNA m^6^A Methylation

The m^6^A levels of RNA of cells were measured using the EpiQuik^TM^ m^6^A RNA Methylation Quantification Kit (#P-9005, Epigentek, Farmingdale, NY, USA), according to the manufacturer’s instructions (Catalog#P-9005-96, EpigenTek) [41,42]. Briefly, total RNA was extracted from cells of MDA-MB-468, MCF-7, MCF-10A cell lines using SV total RNA isolation kits (Promega Corporation, Madison, WI, USA). Extracted RNA (400 ng each) of all samples were utilized in the ELISA assay and quantitated with the m^6^A standard curves, as described previously.

### 2.7. Western Blot Analysis

It was performed as described previously [2,43]. Briefly, treated cells or tissue homogenates were lysed in the NP-40 Surfact-Amps detergent solution (Biosource, Camarillo, CA, USA) to extract total cellular proteins. The sample proteins were measured using a bicinchoninic acid (BCA) protein assay kit (Pierce, Rockford, IL, USA). Equal amounts of proteins (50 µg/lane) were resolved on 4˗20% SDS-PAGE (Thermo Fisher Scientific) and transferred to nitrocellulose membrane. The blocked blots were incubated with each primary antibody (1:100 or 1:5000 dilution; at 4 °C overnight), washed and then incubated with corresponding HRP-conjugated secondary antibody (1:5000 dilutions) or m-IgG1 BP-HRP (1:10,000; cat#, sc-525408). After washing with PBS solution, the blots were incubated with SuperSignal West Femto substrate (Thermo Fisher Scientific) for detection. Glyceraldehyde-3-phosphate dehydrogenase (GAPDH) was applied as an internal control for comparison. Relative levels of proteins, based on optical densities of the blots were assessed with ImageJ (https://imagej.net/ij/) and normalized against GAPDH. Antibody against p53 phosphorylated at Ser15 (rabbit, cat#9284), MICA/B (major histocompatibility complex-I chain-related proteins A and B; rabbit monoclonal, E9N7G) were purchased from Cell Signaling Technology (Danvers, MA, USA). Antibodies for human p53, GAPDH, CCAR2/DBC-1 (cell cycle and apoptosis regulator 2; mouse monoclonal H-2), Bax (mouse monoclonal 6A7), and ULBP1 (UL16-binding protein 1; mouse monoclonal 3F1) and m-IgG1 BP-HRP were purchased from Santa Cruz Biotechnology (Dallas, TX, USA).

### 2.8. Data Analysis

All experiments in present study were carried out in triplicate and repeated at least two times. Data are presented as mean ± SD. On-way or Two-way ANOVA was applied for multiple group comparisons, followed by Tukey’s multiple comparisons test. Prism v10 (GraphPad, San Diego, CA, USA) was used for statistical analyses. A *p*-value < 0.05 between two groups was defined as statistical significance.

## 3. Results

### 3.1. p53-Mutant Cancer Cells Repress NK Cell Proliferation and Decrease NK Cytotoxic Activity to Tumor Organoids

**Figure 1:**
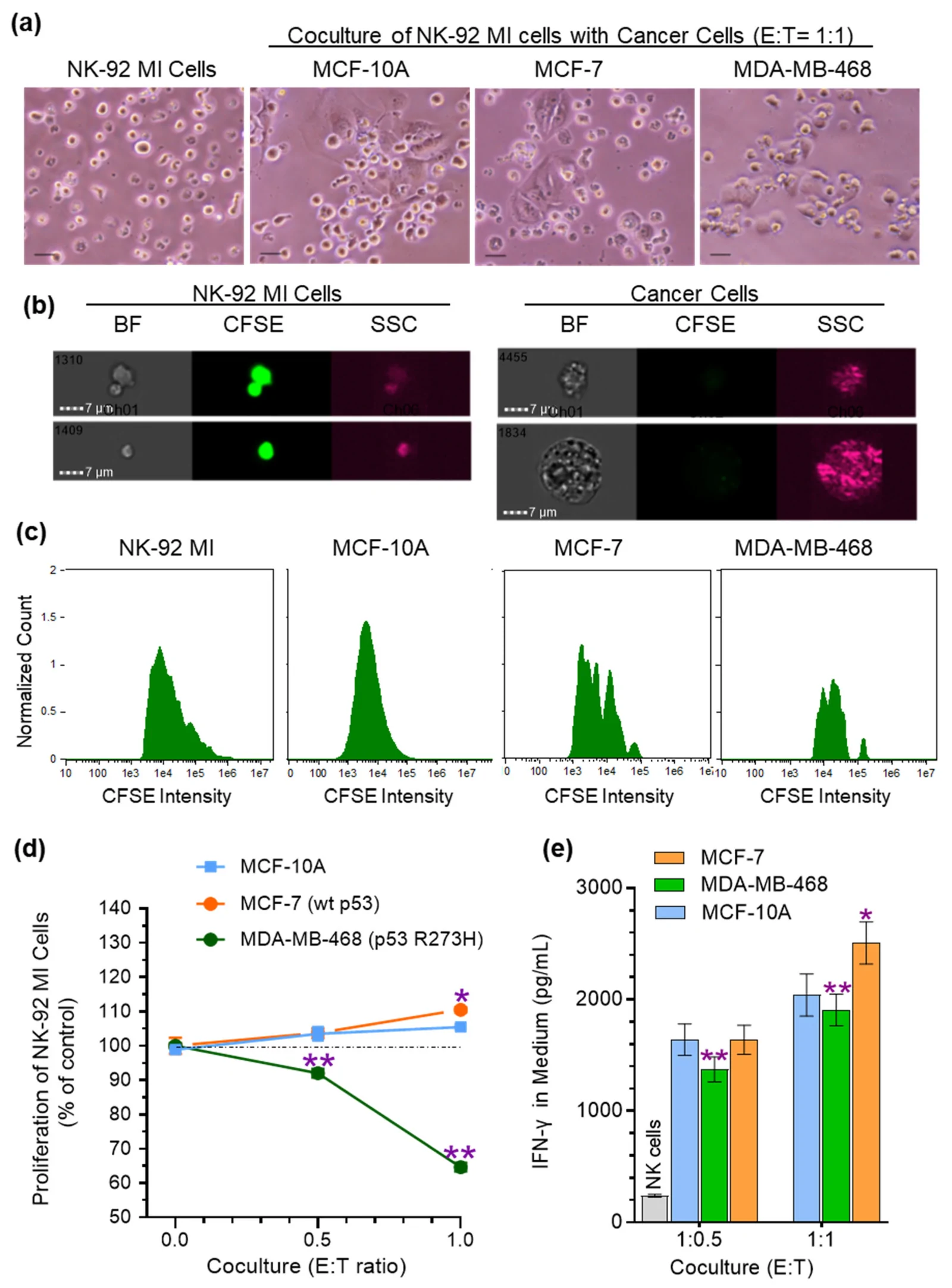
p53 R273H mutant in cancer cells inactivated NK cell proliferation. The CFSE labelled NK-92 MI cells (2.5 × 10^4^ cells) NK culture medium containing 0.2 μM doxorubicin (Dox) were placed to 24-well plates alone or with cancer cells (E/T ratios 1:0.5, 1:1) and co-cultured for 48 h. (**a**) Photomicrographs of cancer cells co-cultured with NK-92 MI cells. Images of co-culture were captured using an EVOS FL cell imaging system with color CCD camera (200× magnification), and scale bar represents 50 μm. (**b**) Imaging flow cytometry for NK-92 MI cells and cancer cells. (**c**) Histogram plots of NK-92 MI cells analyzed using IFC. (**d**) Proliferation of NK-92 MI cells in co-culture with cancer cells. E:T ratio, effector, NK-92 MI cells to targets of cancer cells. Control, NK-92 MI cells alone at the same condition; *, *p* < 0.001 compared to vehicle control; **, *p* < 0.001 compared to MCF-10A or MCF-7 cells. (**e**) IFN-γ levels in co-culture medium. *, *p* < 0.01 compared to MCF-10A cells; **, *p* < 0.001 compared to MCF-7 or MCF-10A cells. Data are expressed as mean ± SD and significance are determined using two-way ANOVA following by Tukey’s multiple comparisons test.

The role of p53 R273H mutated by breast cancer in modulating cancer immunity is still unclear, even knock-in p53 R273H by lung cancer cells or p53 R242Q of breast cancer cells derived from transgenic mice inactivated NK cell-based immunity [10,25]. We directly examined the effects of p53 status by cancer cells on activation of NK-92 MI cells in co-culture. NK-92 MI cells were labeled with CFSE and cultured with cancer cells under doxorubicin (Dox) induced stress of for 48 h, and NK-92 MI cells were assessed by imaging flow cytometry. NK-92 MI cells, which displayed synergistic effects against cancer cells under exposure to subtoxic dose of Dox [44,45], were served as controls for NK cell activation. Under the same conditions, MCF-7 (wt-p53) cancer cells significantly increased the numbers of NK cells in co-culture (E:T = 1:1), as compared to non-tumorigenic MCF-10A cells (Figure 1a–d and Figure S1). In contrast, increasing numbers of MDA-MB-468 cells consistently decreased the numbers of NK cells, by approximately 3-fold (−8% vs. 3.7%, *p* < 0.001; E:T = 1:0.5) and 4-fold (−35.4% vs. 10.5%, *p* < 0.001; E:T = 1:1), respectively vs. MCF-7 controls (Figure 1a–d). Interferon-γ (IFN-γ), pleiotropic cytokine for tumor surveillance, often is mainly produced by NK cells. We further quantitatively analyzed IFN-γ levels released in the co-culture media of NK cells with cancer cells using ELISA. We found that activated NK cells released large amounts of IFN-γ into the media when co-cultured with wtp53 cells (Figure 1e). With increasing cell numbers of MCF-10A or MCF-7 activating NK cell proliferation (Figure 1a–d), the IFN-γ levels in media were increased substantially, displaying a dose-response pattern (E:T, 1: 0.5 to 1:1; Figure 1e). However, mtp53 MDA-MB-468 cells, which were seen to repress NK cell proliferation (cf. Figure 1d), significantly decreased IFN-γ levels in media co-cultured with NK cells, compared to MCF-7 or MCF-10A (Figure 1e).

**Figure 2:**
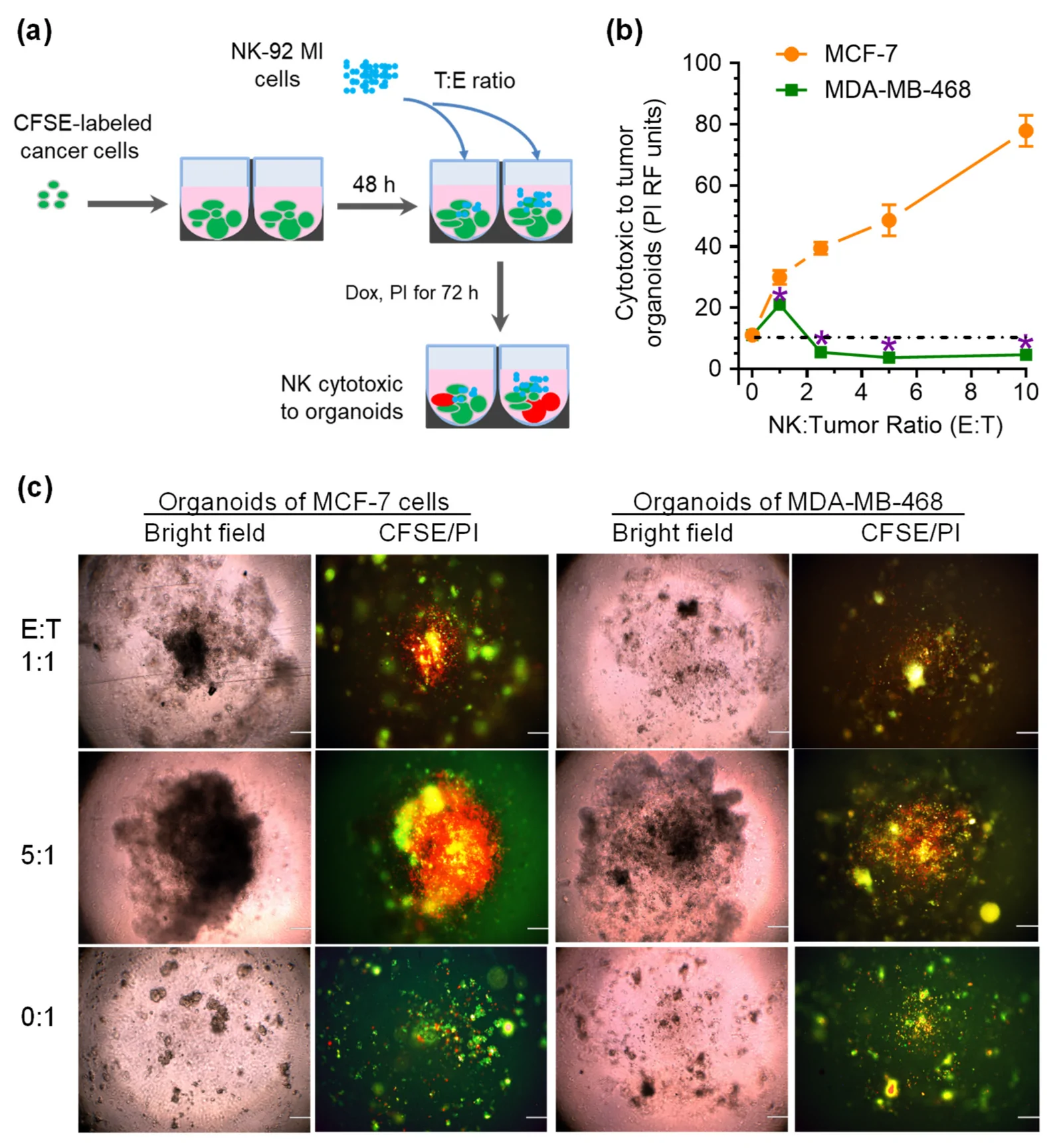
p53 mutant decreased NK cytotoxic effects on tumor organoids. Organoids of MCF-7 and MDA-MB-468 cancer cells (2000/well) were co-cultured with NK-92 MI cells (2000, 2500, 5000, 20,000) in medium containing Dox (0.2 μM) and PI for 72 h. (**a**) Tumor organoids co-cultured with NK cells. (**b**) Cytotoxic activity of NK cells to tumor organoids. *, *p* < 0.001 compared to MCF-7 organoids. (**c**) Representative images of organoids with NK-92 MI cells. Images of NK-organoids were captured using an EVOS FL cell imaging system with color CCD camera (100× magnification; Thermo Fisher Scientific) and scale bar represents 100 μm. Green fluorescence, CFSE-labeled tumor organoid; red fluorescence, PI counterstained died cells. Data are expressed as mean ± SD and significance are determined using two-way ANOVA following by Tukey’s multiple comparisons test.

Further, we evaluated the effects of p53-mutant cancer cells on cytotoxic activity of NK cells in tumor organoids. We added NK-92 MI cells on organoids of cancer cells and measured died organoids after 3 days co-culture (Figure 2a). With increase of additions of NK cells to the organoids of MCF-7 cells, the cytotoxic activity of NK cells displayed a dose-response patterns (Figure 2b,c). In contrast, cytotoxic activities of NK cells significantly decrease, rather than increase in co-cultures with MDA-MB-468 cells (Figure 2b,c). The numbers of died organoids of MDA-MB-468 cells were decreased by 1-fold (11 vs. 19), 6-fold (−5 vs. 28.4), 6-fold (−7.4 vs. 37.6), 11-fold (−6.4 vs. 66.9), compared to MCF-7 cells, respectively (Figure 2b,c). These results indicate that p53 R273H by breast cancer cells deactivated NK cells and decrease its cytotoxic activity against cancer.

### 3.2. Inhibition of m^6^A Methylation in p53 Mutant-Cancer Cells Reactivates NK Cell Proliferation and Increase NK Cytotoxic Activity to Tumor Organoids

**Figure 3:**
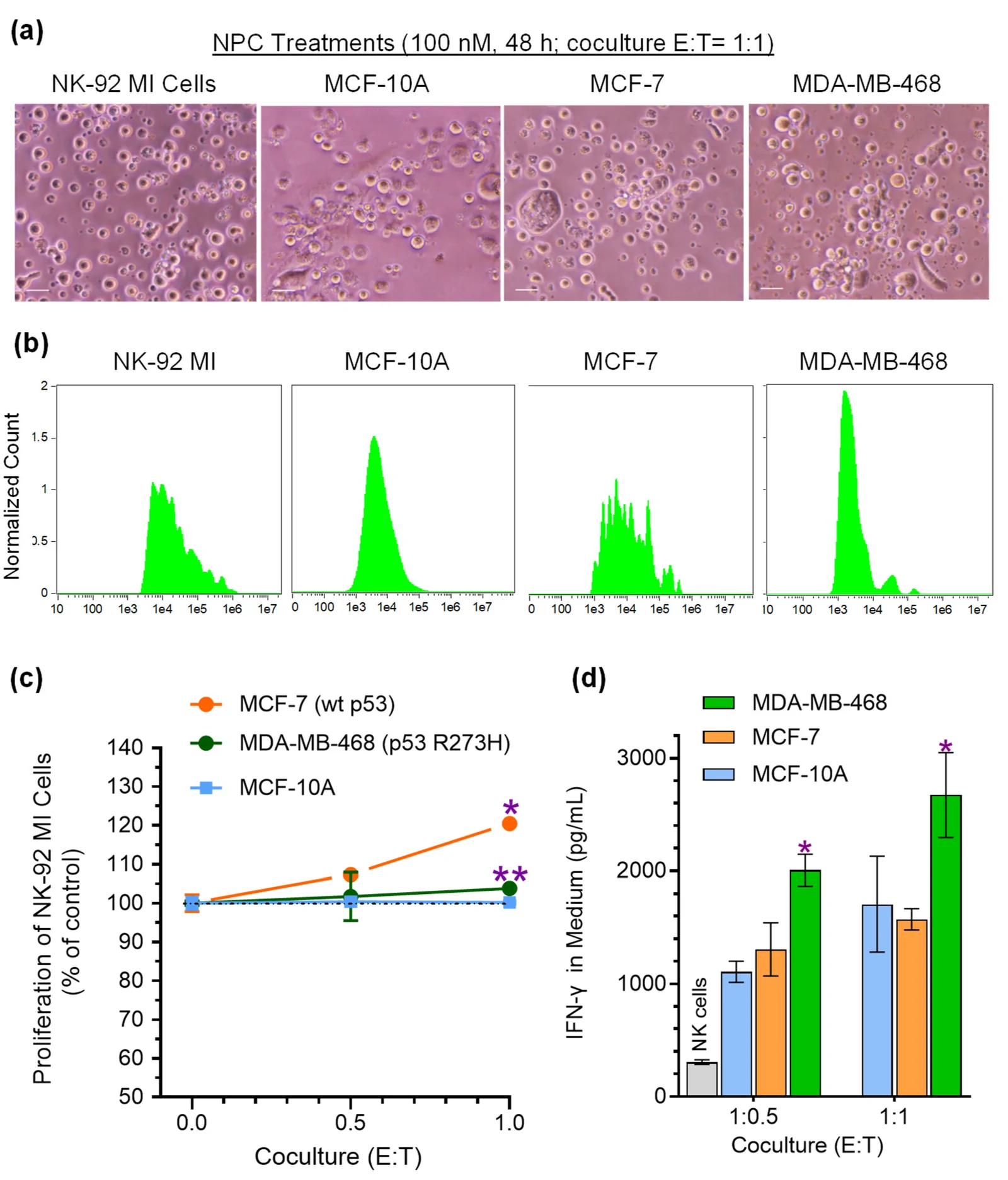
Inhibition of m^6^A methylation in cancer cells activated NK cell proliferation. The CFSE labelled NK-92 MI cells (2.5 × 10^4^ cells) NK culture medium containing 0.2 μM doxorubicin (Dox) and 100 nM NPC were placed to 24-well plates alone or with cancer cells (E/T ratios 1:0.5, 1:1) and co-cultured for 48 h. (**a**) Co-culture of breast cancer cells with NK-92 MI cells. Images of co-culture were captured using an EVOS FL cell imaging system with color CCD camera (200× magnification), and scale bar represents 50 μm. (**b**) Histogram plots of NK-92 MI cells analyzed using IFC. (**c**) Proliferation of NK-92 MI cells in co-culture with cancer cells. E:T ratio, effector, NK-92 MI cells to targets of cancer cells. Control, NK-92 MI cells alone at the same condition; *, *p* < 0.001 compared to vehicle control; **, *p* < 0.001 compared to MCF-10A or MCF-7 cells. (**d**) IFN-γ levels in co-culture medium. *, *p* < 0.01 compared to MCF-7 cells. Data are expressed as mean ± SD and significance are determined using two-way ANOVA following by Tukey’s multiple comparisons test.

Previous studies indicate that m^6^A methylation modulates the GOF of p53 mutants, such as drug resistance in colon cancer cells harboring p53 R273 [2,46,47]. Neplanocin A (NPC), a potent inhibitor of S-adenosylhomocysteine hydrolase [48], can efficaciously inhibit m^6^A methylation by METTL3/14 in colon cancer cells [2]. We further examined the effects of NPC on cancer cells to NK cell activation. MCF-7 (wt p53) cells treated with NPC and Dox, significantly increased the numbers of NK cells in co-culture (E:T = 1:1), as compared to MCF-10A cells (Figure 3a–c and Figure S2). Interestingly, NPC with Dox treatments of MDA-MB-468 cells substantially increased the numbers of NK cells, by approximately 4-fold (7.3% vs. 1.7% at E:T = 1:0.5, *p* < 0.001) and 5-fold (20.5% vs. 3.8% at ET = 1:1, *p* < 0.001), respectively, compared to either MCF-7 cells or MCF-10A cells (Figure 3a–c). Remarkably, inhibition of m^6^A methylation with NPC reactivated NK cells co-cultured with MDA-MB-468 cells treated with NPC and Dox, the numbers of NK cells increased by approximately 2-fold (7.3% vs. −8% at E:T = 1:0.5; 20.5 vs. −35.4% at E:T = 1:1, *p* < 0.001), compared to MDA-MB-468 cells treated with Dox alone (Figure 1d and Figure 3c). Consequently, reactivated NK cells produced and then released large amounts of IFN-γ into the media when co-cultured with MDA-MB-468 cancer cells treated with NPC and Dox (Figure 3d). With increasing cell numbers of MDA-MB-468 cells (as well as MCF-10A or MCF-7 cells) and activated NK cells (Figure 1a–c), IFN-γ levels released in media substantially increased, displaying a dose-response pattern (E:T, 1: 0.5 to 1:1; Figure 3d). The IFN-γ levels in media of NK cells co-cultured with MDA-MB-468 cells significantly increased after NPC and Dox combined treatments, compared to MCF-7 or MCF-10A (*p* < 0.001, Figure 3d).

**Figure 4:**
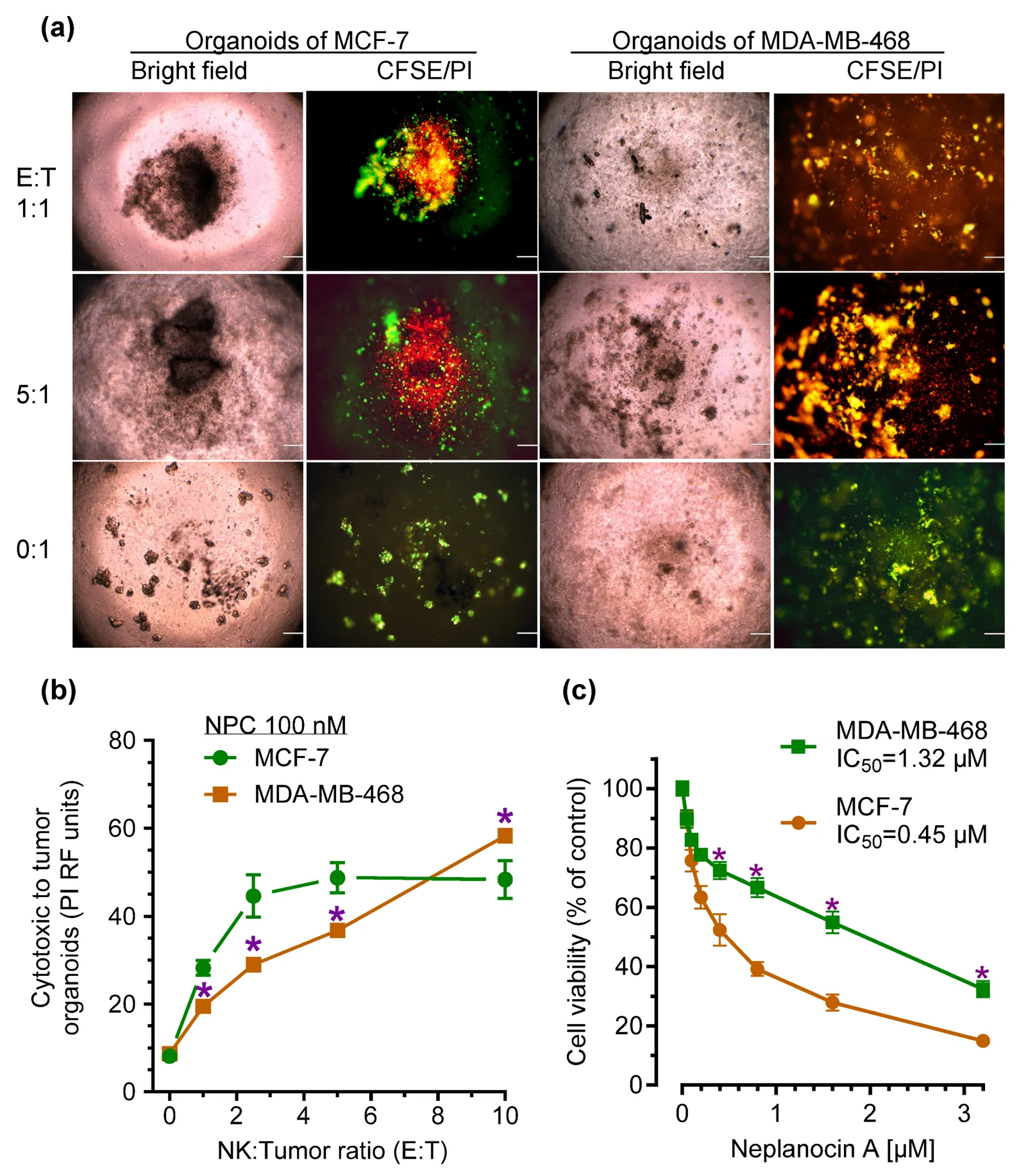
p53 mutant decreased NK cytotoxic effects on tumor organoids. Organoids of MCF-7, and MDA-MB-468 cells (2000/well) treated with neplanocin A (NPC) were co-cultured with NK-92 MI cells (2000, 2500, 5000, 20,000) medium containing Dox (0.2 μM) and PI for 72 h. (**a**) Representative images of organoids with NK-92 MI cells. Images of NK-organoids were captured using an EVOS FL cell imaging system with color CCD camera (100× magnification; Thermo Fisher Scientific) and scale bar represents 100 μm. Green fluorescence, CFSE-labeled tumor organoid; red fluorescence, PI counterstained died cells. (**b**) NPC effects on tumor organoids co-cultured with NK cells. (**c**) Cell viability for NPC. Cells were cultured in 5% FBS medium containing indicated concentrations of NPC for 72. *, *p* < 0.001 compared to MCF-7 cells exposed to NPC. Data are expressed as mean ± SD and significance are determined using two-way ANOVA following by Tukey’s multiple comparisons test.

With increase of additions of NK cells to tumor organoids treated with NPC and Dox, the cytotoxic activity of NK cells displayed a dose-response patterns (E:T 0.5 to 5) (Figure 4b). Furthermore, cytotoxic activities of NK cells significantly increased in NK co-cultured with MDA-MB-468 cells treated with NPC (Figure 2b and Figure 4b). The numbers of died organoids of MDA-MB-468 cells were increased by approximately 5-fold (5.4 vs. 29; E:T = 2.5:1), 10-fold (36.8 vs. 3.6; E:T = 5:1) and 13-fold (58.3 vs. 4.6; E:T = 10:1), compared to MDA-MB-468 cells treated with Dox alone, respectively (Figs 2b, 4b). The IC_50_ values for NPC in cell viability are approximately 0.5 μM and 1.3 μM in MCF-7 and MDA-MB-468 cells, respectively (Figure 4c). These results indicate that inhibition of m^6^A methylation of p53-mutant cancer cells reactivated NK cells and increase NK cytotoxic activity against cancer.

### 3.3. RNA m^6^A Modification Modulates the Expression of p53 R273H in Cancer Cells and Deactivates NK Cells Through Decreased Expression of NKG2D Ligands

**Figure 5:**
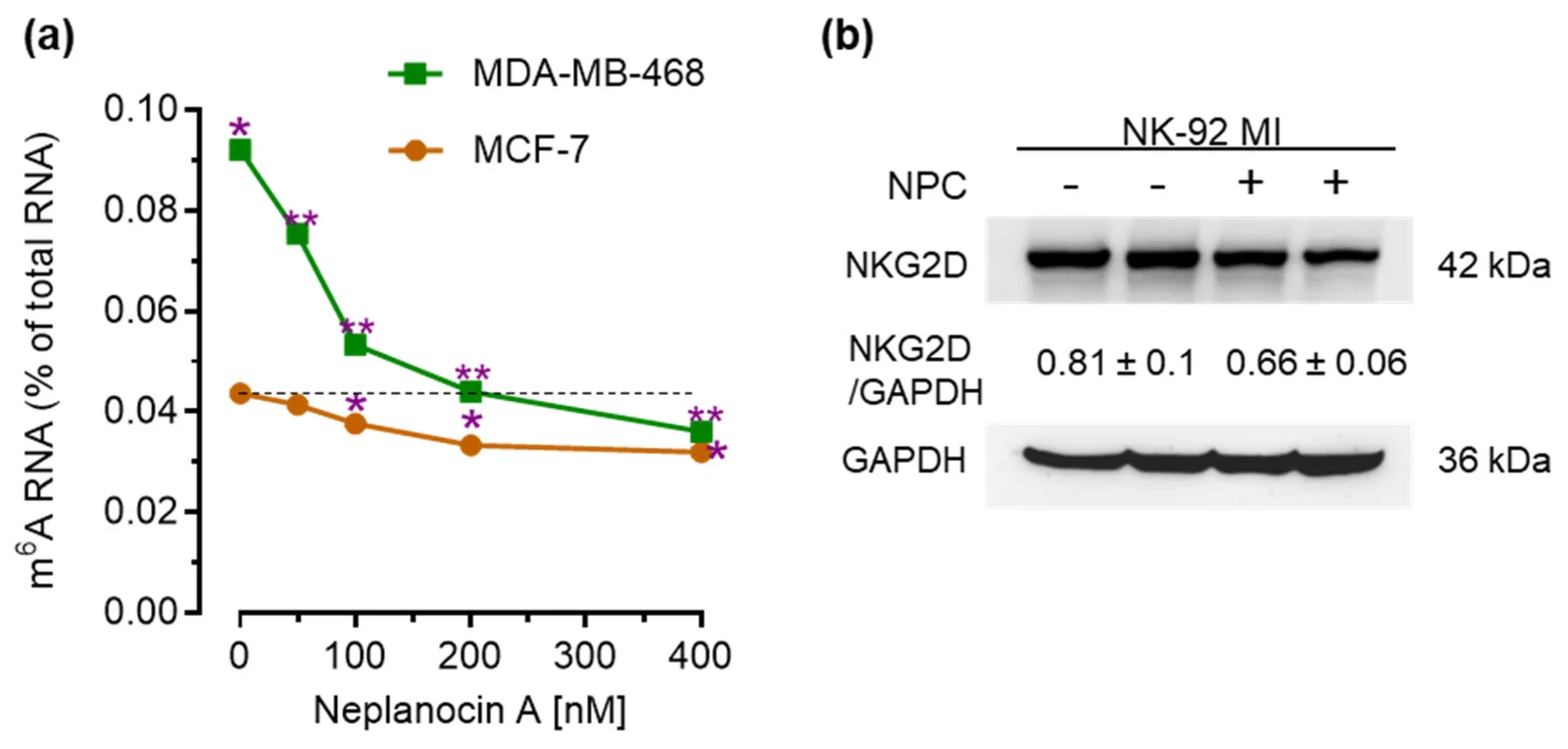
NPC effects on m^6^A methylation and cell viability. (**a**) Cell viability for NPC. NPC treatments inhibited m^6^A RNA methylation of cancer cells. Cells were cultured in 5% FBS medium containing Dox (0.2 μM) alone or combined with NPC for 48 h. Levels of m^6^A-RNA were quantitated with EpiQuik^TM^ m^6^A methylation quantification kit. *, *p* < 0.001 compared to MCF-7 cells exposed to Dox. **, *p* < 0.001 compared to MDA-MB-468 cells exposed to Dox alone. (**b**) Western blotting of NKG2D receptor in NK-92 MI cells. NK-92 MI cells were treated with Dox (0.2 μM) alone or combined with NPC (100 nM) for 48 h. Equal amounts of extracted proteins (50 µg/lane) were resolved by SDS-PAGE and immunoblotting. Data are expressed as mean ± SD and significance are determined using two-way ANOVA following by Tukey’s multiple comparisons test.

We examined the effects of NPC on m^6^A RNA methylation of breast cancer cells. With quantitative analysis with ELISA of cells exposed to Dox, we found that the m^6^A-RNA in MDA-MB-468 cells that carrying p53R273H (-Cm^6^AU-) is approximately 2-fold higher than (0.09% vs. 0.04%, *p* < 0.001) these in MCF-7 cells (codon 273 -CGU-) (Figure 5a). NPC treatments at nanomole-range significantly reduced the m^6^A levels in dose-response manner, particularly for MDA-MB-468 cells exposed to Dox and the IC_50_ values is 68 nM, compared with 106 nM in MCF-7 cells (Figure 5a). NKG2D receptors that can be activated with NKG2D ligands have been reported presenting in NK cells. NK-92 MI cells expressed NKG2D receptor under the treatments of Dox alone or combined with NPC (Figure 5b).

**Figure 6:**
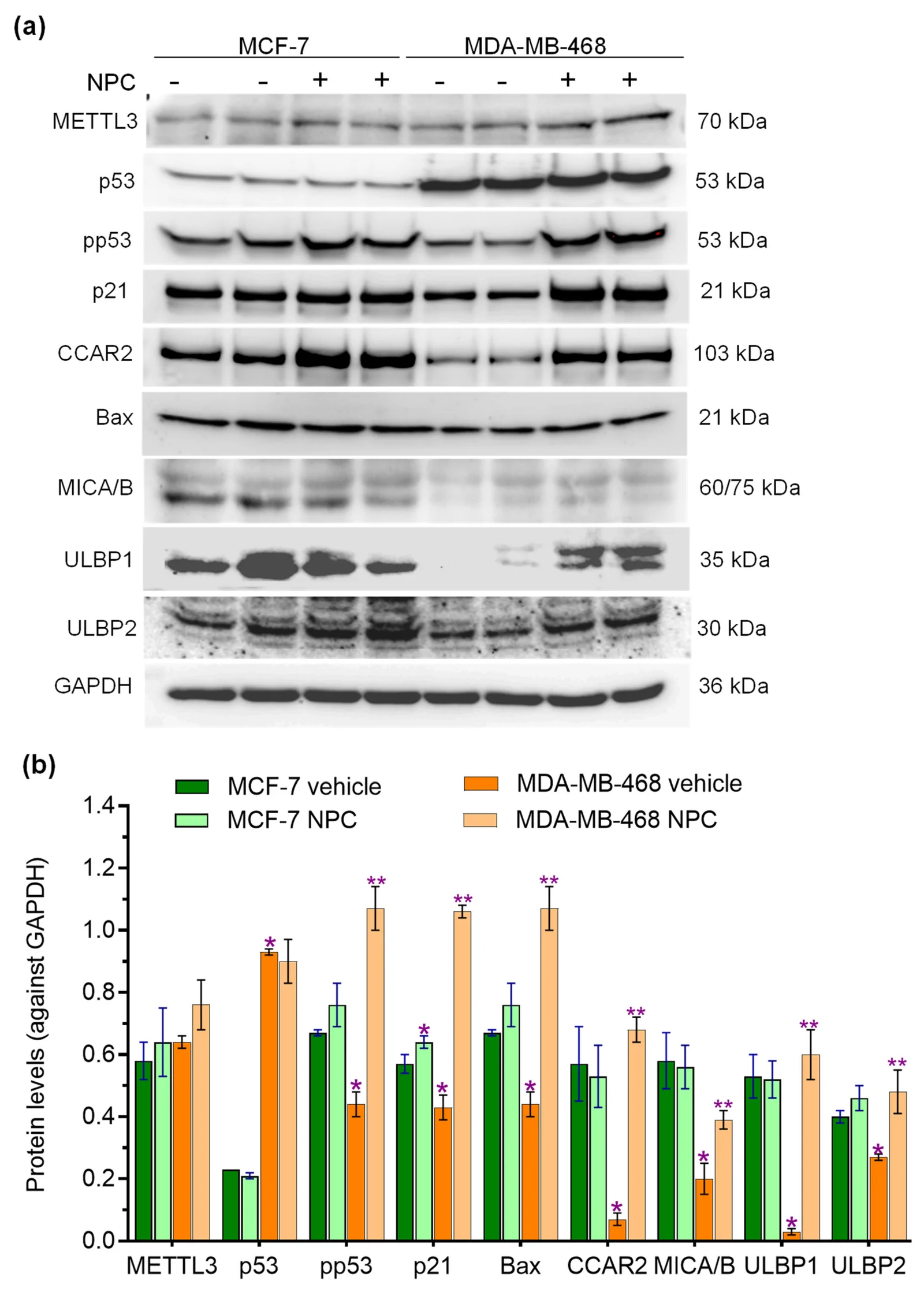
m^6^A methylation at p53R273 regulated the expression of p53 and NKG2D ligands by cancer cells to activate NK cells. (**a**) Western blotting of p53-responsive proteins. Cells were exposed to Dox (0.2 μM) alone or combined with NPC (100 nM) for 48 h. Equal amounts of extracted proteins (50 µg/lane) were resolved by SDS-PAGE and immunoblotting. (**b**) Relative levels of p53-associated proteins and NKG2D ligands. *, *p* < 0.001 compared to MCF cells exposed to Dox alone; **, *p* < 0.001 compared to MDA-MB-468 cells exposed to Dox alone. Data are expressed as mean ± SD and significance are determined using two-way ANOVA following by Tukey’s multiple comparisons test.

Under the same conditions, we assessed the correlation of expression of p53-associated NKG2D ligands by cancer cells to NK cell activation. In conjunction with production of phosphorylated p53 and p53-associated proteins (CCAR2, Bax), MCF-7 (wtp53) cells either exposed to Dox or Dox with NPC, expressed substantial levels of NKG2D ligands, including MICA/B and ULBP1 that can activate the NKG2D receptors of NK-92 MI (Figure 6a,b). MDA-MB-468 cells expressed lower levels of pp53, although the levels of total p53 levels were significantly higher than MCF-7 cells (Figure 6a,b). Consistence with CCAR2 and Bax, the levels of MICA/B and ULBP1 of MDA-MB-468 cells are substantial lower, by approximately 3-fold (0.2 vs. 0.6, *p* < 0.001) and 17-fold (0.03 vs. 0.5, *p* < 0.001), respectively, compared with these of MCF-7 Cells (Figure 6a,b). Furthermore, NPC treatments significantly increased the levels of pp53 of MDA-MB-468 cells, by approximately 2-fold (0.4 vs. 1.1, *p* < 0.001), compared with Dox alone treatments (Figure 6a,b). Consequently, the p53-associated protein levels in MDA-MB-468 cells treated with Dox and NPC, including Bax and CCAR2 enhanced by approximately 2-fold (0.4 vs. 1.1, *p* < 0.001) and 6-fold (0.1 vs. 0.7, *p* < 0.001), respectively (Figure 6a,b). The levels of NKG2D ligands, including MICA/B and ULBP1, are increased by approximately 2-fold (0.2 vs. 0.4, *p* < 0.001) and 20-fold (0.03 vs. 0.6, *p* < 0.001) respectively in MDA-MB-468 cells treated Dox and NPC, compared to Dox alone treatments. These results indicate inhibition of m^6^A modification with NPC can increase expression of activating NKG2D ligands in cancer cells to activate NK-92 MI cells.

## 4. Discussion

p53 serves as a guardian of immune integrity, regulating innate and adaptive immunity to suppress tumorigenesis. However, as arrogated oncogenic proteins, p53 mutants in tumors can promote immune evasion and tumor progression by modulating the productions of exosomes, growth factors, cytokines and chemokines [6,49].p53 mutants in cancer cells can directly interact with immune cells, such as NK cells and modulate immune evasion [10,23]. Our present study with evidence in cell-cell interaction and molecular mechanism elucidated that p53 R273H of cancer cells deactivates NK cells to immune evasion. In co-culture with NK-92 MI cells, p53 in MCF-7 cancer cells, can activate NK cells and induce higher levels of IFN-γ than noncancer MCF-10A, thus destructing tumor organoids (Figure 1 and Figure 2). However, MDA-MB-468 cells that carry p53 R273H mutant deactivated NK cells, and decreased production of IFN-γ levels, further their tumor organoids evaded destruction (Figure 2). These results are consistence with the distinct effect of wild-type p53 and Trp53 G242A on NK cells in immune evasion [10,23].

**Figure 7:**
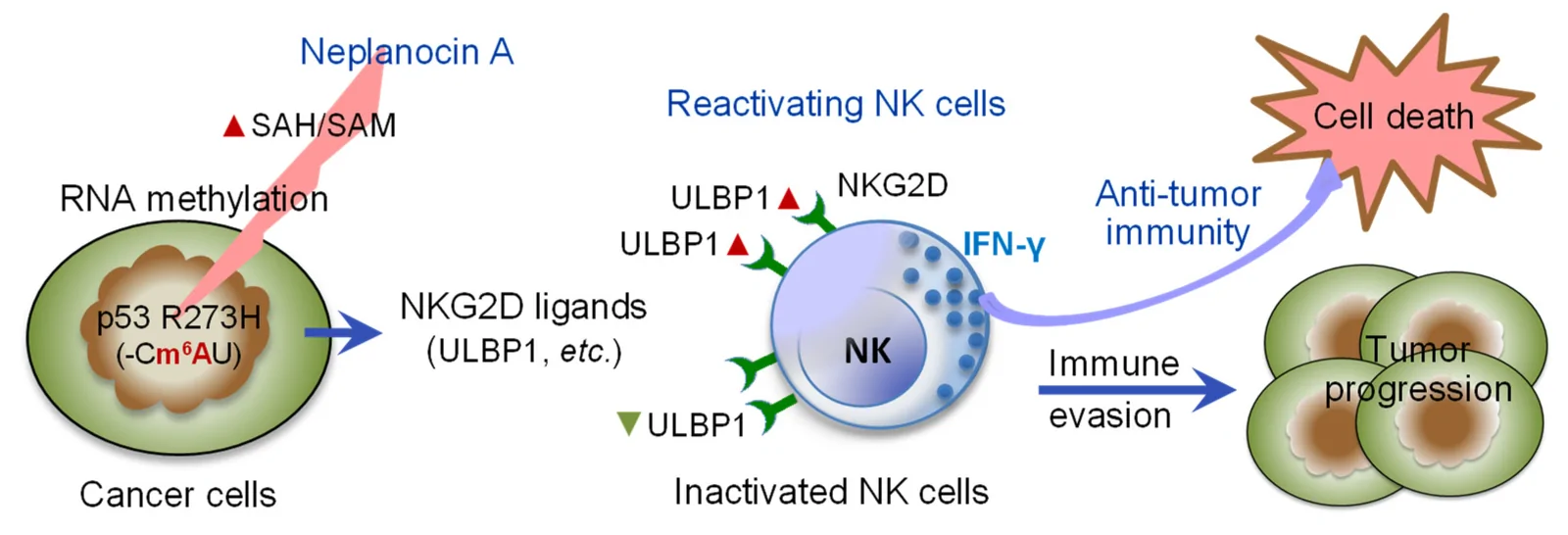
Inhibition of RNA m^6^A methylation in p53-mutant cancer cells reactivates NK cells to destruct tumor. Mutant proteins from *TP53* R273H modulate the repressed expression of NKG2D ligands, including ULBP1, ULPB2, and MICA/B, and inactivate NK cells, thus facilitating immune evasion and tumor progression. Suppression of RNA m^6^A methylation by neplanocin A (NPC), which inhibits *S*-adenosylhomocysteine (SAH) hydrolase and disrupts SAH/SAM ratio for methyl group transfer, upregulates the expression of p53-associated NKG2D-ligands (ULBP1, etc.), reactivating NK cells and immune destruction against tumor. ▲ SAH, increased levels of SAH; SAM, *S*-adenosylmethionine; ▼ or ▲, repressed or increased expression of UPLBP1 and other NKG2D ligands.

RNA m^6^A methylation in response to stress can mediate mRNA formation and stability and adapts cancer cells resistance to treatments [50,51]. Recent report showed that RNA m^6^A modification by KRAS mutation drives immune evasion in colorectal cancer [51]. However, METTL3-mediated m^6^A RNA methylation also has been reported to promote the anti-tumor immunity of NK cells [52]. Our present study elucidated that inhibition of RNA m^6^A methylation can argument NK cells-based tumor immunity against p53 R273H-cancer cells. NPC displayed as an efficacious inhibitor of RNA m^6^A methylation in mutant cancer cells, particularly in p53 R273H cells of MDA-MB-468 (Figure 5a). Inhibition of RNA m^6^A methylation with NPC remarkably activated NK cell proliferation and IFN-γ production in co-cultures (Figure 1d and Figure 3c), and augmented cytotoxicity of NK cells to tumor organoids by 5 to 13-fold (Figure 2b and Figure 4b). Compared to wild-type p53 (-CGU), p53 R273H (-CAU-) of cancer cells can be methylated as (-Cm^6^AU-) by METTL3 [2] (Figure 7). Acting as specific inhibitor of *S*-adenosylhomocystein (SAH) hydrolase, NPC can suppress methylation reaction in RNA or DNA, via disrupting SAH/SAM ratio and competitive inhibiting *S*-adenosylmethionine (SAM) to methyltransferases [53,54] (Figure 7). NPC treatments in nanomole range significantly reactivated NK cell-based anticancer immunity, suggests inhibition RNA m^6^A modification with NPC is a potential approach for reactivating NK cells against immune evasion with cancer cells carrying missense mutants that can be methylated to m^6^A, such as p53R273H. With its broader inhibitory impacts on cellular methylation pathways, NPC may result in unanticipated adverse effects on normal tissues. Previous study showed that NPC-induced cytotoxicity in Chinese hamster ovary cells, via depletion of cellular ATP and RNA and protein synthesis [55]. Further in vivo studies are required to disclose NPC effects on tumors as well as on normal tissues and immune responsiveness.

p53 can stimulate the expression of the NKG2D ligands (ULBP1, MICA/B, ICAM-1 in cancer cells to activate NK cells in immunosurveillance of senescent tumors [23,24,56]. Gain-of-function p53 can lead tumor immune evasion by downregulating the expression of NKG2D ligands, ULBP1/2 on tumor cells [23]. In present study, MDA-MB-468 cells exhibited lower levels of pp53, and also lower levels of MICA/B and ULBP1, compared to MCF-7 cells (Figure 6a,b). Inhibition of m^6^A methylation with NPC enhancing the expression levels of pp53 and p53-associated genes (p21, Bax, CCAR2) in MDA-MB-468 cells with accumulated levels of ULBP1 and MICA/B, compared to these cells treated with Dox alone (Figure 6a,b). These results further indicate the distinct effects of p53 and missense mutant p53 on NKG2D ligands expression in cancer cells and also proves that inhibition of m^6^A methylation of MDA-MB-468 cells strengthens p53 dependent-expression of stimulating NKG2D ligands (ULBP1, MICA/B), thus reactivate NK cells. Altogether, this study demonstrated that inhibition m^6^A methylation with NPC provides an effective approach for targeting immune evasion in p53 mutant cancer.

## Data Availability

The original contributions presented in this study are included in the article and Supplementary Materials. Further inquiries can be directed to the corresponding author.

## Supplementary Materials

The following supporting information can be downloaded at: https://www.mdpi.com/article/10.3390/cimb48101021/s1.

## Abbreviations

The following abbreviations are used in this manuscript:

NPC	Neplanocin A
NK	Natural killer cells
IFN-γ	Interferon-gamma
Dox	Doxorubicin
m^6^A	*N^6^*-methyladenosine
NKG2D	Natural killer group 2D receptor
mtp53	Mutant p53
wtp53	Wild-type p53
pp53	Phosphorylated p53 protein
GOF	Gain-of-function
IC_50_	Half-maximal inhibitory concentration
p21	P21 protein also called Cip1/Waf1
Bax	BCL2-associated X protein
CCAR2	Cell cycle and apoptosis regulators 2
MICA/B	Major histocompatibility complex (MHC) class I chain-related protein A and B
ULBP1	UL16 binding protein 1,
