# Supplementary material for: Inhibition of RNA-m6A Methylation in Breast Cancer Cells Carrying TP53 R273H Mutant Reactivates Natural Killer Cells for Anticancer Immunity
Source: Curr Issues Mol Biol. 2026 Oct 2;48(10):1021. doi: 10.3390/cimb48101021 (PMC13703405; doi:10.3390/cimb48101021)
Supplement: Supplementary file 1 [file cimb-48-01021-s001.zip › cimb-4519885-supplementary.pdf]

Supplementary

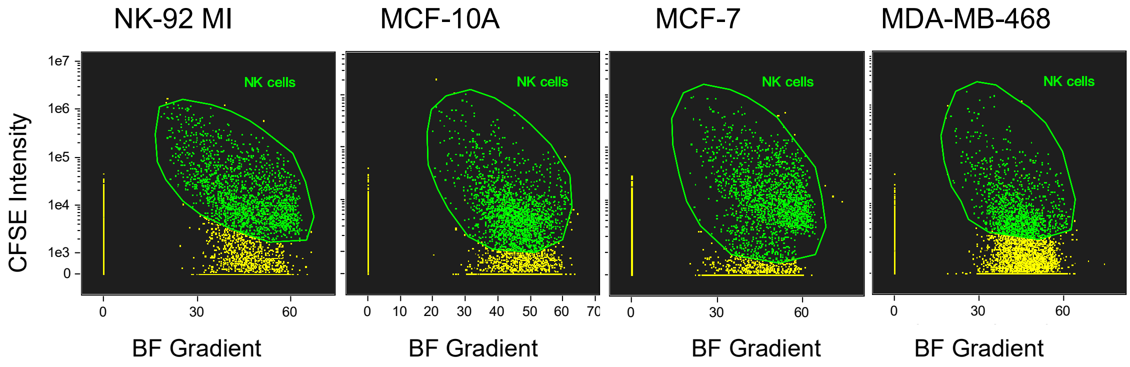

Figure S1. NK-92 MI cells analyzed using IFC. The CFSE labelled NK-92 MI cells (2.5 × 10^4 cells) NK culture medium containing 0.2 μM doxorubicin (Dox) were placed to 24-well plates alone or with cancer cells (E/T ratios 1:1) and co-cultured for 48 h.

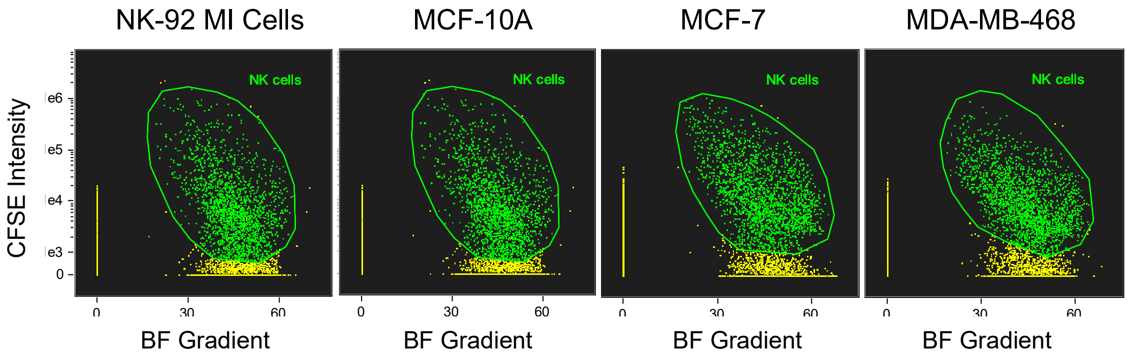

Figure S2. NK-92 MI cells analyzed using IFC. The CFSE labelled NK-92 MI cells (2.5 × 10^4 cells) in NK culture medium containing 100 nM NPC and 0.2 μM doxorubicin (Dox) were placed to 24-well plates alone or with cancer cells (E/T ratios 1:1) and co-cultured for 48 h.
